# A Common Cancer Risk-Associated Allele in the *hTERT* Locus Encodes a Dominant Negative Inhibitor of Telomerase

**DOI:** 10.1371/journal.pgen.1005286

**Published:** 2015-06-08

**Authors:** Anagha Killedar, Michael D. Stutz, Alexander P. Sobinoff, Christopher G. Tomlinson, Tracy M. Bryan, Jonathan Beesley, Georgia Chenevix-Trench, Roger R. Reddel, Hilda A. Pickett

**Affiliations:** 1 Telomere Length Regulation Group, Children’s Medical Research Institute, University of Sydney, Westmead, New South Wales, Australia; 2 Cell Biology Unit, Children’s Medical Research Institute, University of Sydney, Westmead, New South Wales, Australia; 3 Department of Genetics, QIMR Berghofer Medical Research Institute, Brisbane, Queensland, Australia; 4 Cancer Research Unit, Children’s Medical Research Institute, University of Sydney, Westmead, New South Wales, Australia; Dana Farber Cancer Institute, UNITED STATES

## Abstract

The *TERT-CLPTM1L* region of chromosome 5p15.33 is a multi-cancer susceptibility locus that encodes the reverse transcriptase subunit, hTERT, of the telomerase enzyme. Numerous cancer-associated single-nucleotide polymorphisms (SNPs), including rs10069690, have been identified within the hTERT gene. The minor allele (A) at rs10069690 creates an additional splice donor site in intron 4 of hTERT, and is associated with an elevated risk of multiple cancers including breast and ovarian carcinomas. We previously demonstrated that the presence of this allele resulted in co-production of full length (FL)-hTERT and an alternatively spliced, INS1b, transcript. INS1b does not encode the reverse transcriptase domain required for telomerase enzyme activity, but we show here that INS1b protein retains its ability to bind to the telomerase RNA subunit, hTR. We also show that INS1b expression results in decreased telomerase activity, telomere shortening, and an increased telomere-specific DNA damage response (DDR). We employed antisense oligonucleotides to manipulate endogenous transcript expression in favor of INS1b, which resulted in a decrease in telomerase activity. These data provide the first detailed mechanistic insights into a cancer risk-associated SNP in the *hTERT* locus, which causes cell type-specific expression of INS1b transcript from the presence of an additional alternative splice site created in intron 4 by the risk allele. We predict that INS1b expression levels cause subtle inadequacies in telomerase-mediated telomere maintenance, resulting in an increased risk of genetic instability and therefore of tumorigenesis.

## Author Summary

Multiple cancer-associated single nucleotide polymorphisms (SNPs) associated with risk of a wide variety of cancers have been identified in the *TERT-CLPTM1L* region of 5p15.33, identifying this as a multi-cancer susceptibility locus. *hTERT* encodes the catalytic subunit of the enzyme telomerase, which is responsible for telomere length maintenance in the germline and in most immortalised cancer cells. To date, very little is known regarding the mechanisms by which specific *hTERT* SNPs predispose to cancer. In this study, we carried out detailed functional analyses on the intron 4 SNP rs10069690, which is associated with a small, but highly significant risk for many types of cancer. We show that the risk-associated minor allele of this SNP results in an hTERT mRNA splice variant, encoding a catalytically inactive protein which acts as a dominant negative inhibitor of telomerase activity and therefore decreases total telomerase activity. We propose that individuals who carry the rs10069690 minor allele have less telomerase activity in some cell types due to cell type-specific alternative splicing, which may result in slightly shorter telomeres, and hence an increased risk of genetic instability and tumorigenesis.

## Introduction

Telomeres are nucleoprotein structures, which protect the ends of linear chromosomes from being recognized as DNA double-strand breaks [1]. Telomeres shorten with each round of cell division due to the end-replication problem. Normal human somatic cells replicate until their telomeres diminish to a critical threshold, at which point they enter permanent cell cycle arrest and are constrained to a senescent state [2]. Bypass of senescence due to loss of function of the p53 and pRB tumor suppressor pathways results in further telomere shortening which eventually becomes catastrophic, causing end-to-end fusions, genetic instability, and the potential for tumorigenesis.

Telomere shortening may be counteracted by telomerase, a ribonucleoprotein enzyme complex that synthesizes the repetitive telomeric DNA sequence (5'-TTAGGG-3') [3]. The subunits of telomerase include a reverse transcriptase protein, TERT, and an RNA molecule, hTR, which contains a template region. Telomerase activity is detectable during human development from the blastocyst stage to 16–18 weeks gestation in specific tissue types, but is undetectable in most tissues by two months post-natal [4]. In healthy adults, telomerase activity is restricted to germline cells (in the testes and ovaries) [4], peripheral blood mononuclear cells [5,6] and stem cells [7], presumably to support the proliferative requirements of these cell types. In germline cells, there is sufficient telomerase activity to prevent telomere shortening, but in somatic cells the level of telomerase activity is limited, and is only sufficient to slow down the telomere attrition that accompanies normal DNA replication. In contrast, in the great majority of cancers and immortalized cell lines telomere length is maintained; in 85% of cancers this is due to upregulated levels of telomerase and in the remainder this is due to a non-telomerase mechanism [8,9].

One of the ways in which telomerase activity levels appear to be regulated is via alternative splicing of the TERT pre-mRNA [10]. The TERT gene contains 16 exons, and the TERT pre-mRNA can be spliced to yield more than 20 variant mRNAs [11–13]. During human development, loss of telomerase activity in somatic cells is associated with a change in the TERT splicing pattern such that the transcriptional output of the TERT gene consists entirely of splice variants that do not encode active TERT protein [14,15]. Control of alternative splicing is incompletely understood, but there is evidence that this may involve RNA:RNA pairing within the TERT pre-mRNA [16], and that it may be regulated by the cellular microenvironment [17]. Some of the splice variants encode proteins which can act as dominant negative inhibitors of telomerase activity [18,19]. This may be due in part to their ability to become incorporated into the telomerase enzyme complex, because biochemical studies have demonstrated that telomerase exists as a dimer and that its activity is dependent on both of the hTERT active sites being functional [20].

Mutations in hTERT, and in other genes that are required for normal telomere function, can cause short telomere syndromes, which are characterized by proliferative failure in various tissues, especially the bone marrow, lungs, and liver [21,22]. Patients suffering from these syndromes have a substantially increased risk of cancer [22,23], presumably due to excessive telomere shortening and an increased risk of genetic instability. In contrast to the high risk associated with these relatively rare mutations, genome-wide association studies (GWAS) have uncovered numerous single nucleotide polymorphisms (SNPs) in, or close to the hTERT gene which are relatively common in the population and are associated with a small, but highly significant increase in cancer susceptibility ([13,24–27]; reviewed in [28]). Associations have also been found between *hTERT* SNPs and telomere length [13,24,29].

Despite the statistical robustness of GWAS, the associations remain observational. This is partly because most GWAS have only reported associations with the tagging SNPs in the *TERT-CLPTM1L* region on the commercial genotyping chips, but fine mapping is required to identify the putative causal SNPs. Mechanistic analysis is required to identify causal rather than correlated variants, and to accurately determine cancer risk. A functional understanding of causative variants may ultimately influence clinical decision-making.

Through our involvement with the Collaborative Oncologic Gene-Environment Study (COGS), we were able to carry out fine mapping of the *hTERT* locus. This study revealed that the minor alleles at rs10069690 and at rs2242652 were candidate causal variants for risk of estrogen receptor-negative breast cancer, breast cancer in *BRCA1* mutation carriers, serous low malignant potential ovarian cancer and serous invasive ovarian cancer with odds ratios ranging from 1.15–1.40 [13,24] and for risk of prostate cancer [13,24]. This rs10069690 SNP is also associated with risk of a wide variety of cancers, including colon cancer, acute lymphoblastic leukemia (ALL), and chronic lymphocytic leukemia (CLL) [25,30–32]. However, no fine mapping has been performed for the latter associations to determine whether it is likely to be directly responsible or simply correlated with a better candidate causal SNP. Consequently, there is a growing need to delineate how this allele confers cancer risk.

**Fig 1:**
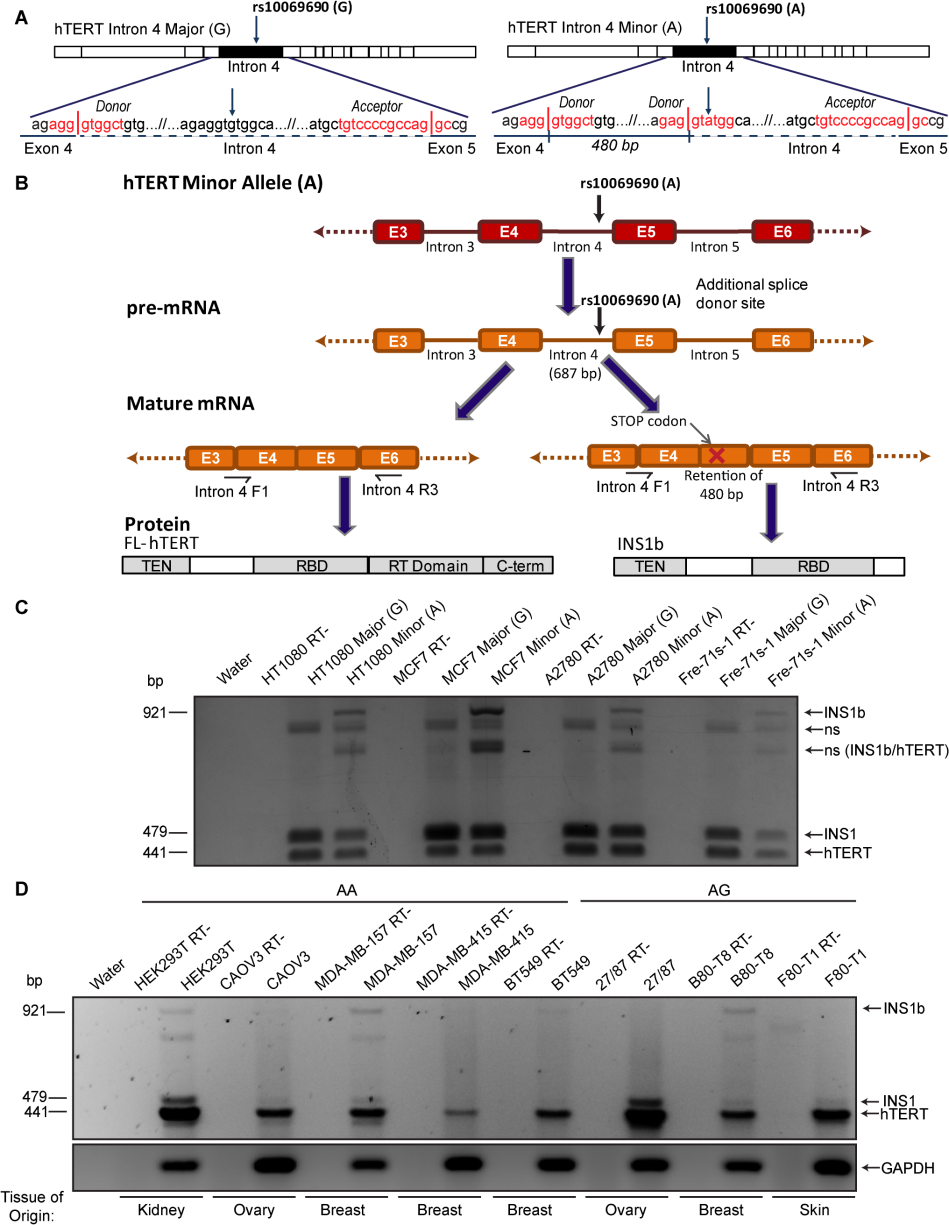
Cancer-associated rs10069690 minor allele results in alternative splicing of hTERT. (A) Schematics of hTERT minigenes generated by inserting the intron 4 sequence, containing either the major (G) or minor (A) allele at rs10069690, into an hTERT cDNA expression construct. The sense-strand sequence of the minigene from the end of exon 4 to the beginning of exon 5 is shown. The red text indicates the splice site consensus sequences with vertical lines representing the exon-intron boundaries. The minor allele at rs10069690, a G to A polymorphism, introduces an additional splice donor site into the sequence. If this site is utilized, 480 bp of intron 4 is retained within the mature mRNA. (B) Schematic of mRNA and protein expression resulting from the minor allele at rs10069690. RT-PCR using primers spanning from the end of exon 3/beginning of exon 4 to the middle of exon 6 generate a 441 bp product from canonically spliced hTERT mRNA, and products that are 38 bp and 480 bp larger from alternative hTERT splice products INS1 and INS1b, respectively. The alternative splice variant formed from the utilization of the additional splice donor site contains a premature stop codon and is therefore predicted to encode a truncated form of hTERT, which contains the telomerase N-terminal (TEN) and RNA binding (RBD) domains, but not the reverse transcriptase (RT) and C-terminal (C-term) domains. (C) Three cell lines (HT1080, MCF7 and A2780) and cell strain (Fre-71s-1) were transfected with hTERT minigenes containing intron 4 with either the Major (G) or Minor (A) allele of rs10069690. RT-PCR was performed with the primers indicated in Fig 1B and products were separated by gel electrophoresis. In each case, the minigene with the minor allele at rs10069690 resulted in co-expression of canonical hTERT, and the variants INS1 and INS1b. Other bands were identified as non-specific (ns) hybrid DNA species, including a mixture of INS1b and canonical hTERT (INS1b/hTERT) transcripts. (D) RT-PCR was performed on cell lines homozygous (AA) or heterozygous (AG) for the minor allele at rs10069690 to identify endogenous expression of INS1b.

We previously generated hTERT minigenes, which included intron 4 with the major (G) or minor, cancer-risk associated (A) allele at rs10069690 [13]. Transfection of these minigenes into the telomerase-positive breast cancer cell line MCF7, and RT-PCR using primers spanning intron 4, revealed an additional band in the presence of the A allele [13]. When this band was excised and sequenced it was identified as a novel hTERT splice variant, INS1b, which contains an intron 4 insert of 480 base pairs. Sequence analysis showed that this transcript was the product of an alternative splicing event at an additional splice donor site created by the G to A polymorphism at rs10069690 (Fig 1A).

In the present study, we investigated the mechanism by which the SNP confers elevated cancer risk. We found that the presence of this polymorphism resulted in the production of both full length and an alternatively spliced hTERT transcript, which were expressed in a cell type-specific manner. The alternative transcript produced a severely truncated protein (INS1b), which retained its hTR binding capability but lacked the reverse transcriptase domain. INS1b directly caused decreased telomerase activity, telomere shortening, and an elevated telomere-specific DNA damage response. Our data demonstrate that INS1b binds to hTR, sequestering it in an inactive form, thereby acting in a dominant negative manner to limit the amount of active telomerase available to extend the telomeres. The combination of the fine mapping data and a mechanism for cancer risk make it highly likely that this is a causal, rather than a correlated cancer risk SNP.

## Results

### Presence of the minor allele at rs10069690 results in the co-production of full length (FL) hTERT and INS1b transcript

Intron 4 minigene constructs with the Major (G) or Minor (A) alleles of rs10069690 were transfected into three telomerase-positive cell lines (HT1080, MCF7 and A2780) and a fibroblast cell strain (Fre-71s-1). The INS1b transcript is 480 nucleotides larger than the canonically spliced transcript, and includes a premature stop codon in the retained intron 4 sequence (Fig 1B). The truncated protein is predicted to retain the N-terminus, TEN domain and hTR binding domains of hTERT, and to lack the reverse transcriptase domain (Fig 1B), and consequently it is expected to be catalytically inactive. Regardless of which allele was present, all cell lines produced a band that was 38 bp larger than the canonical TERT transcript, corresponding to the INS1 splice variant which results from retention of the first 38 bp of intron 4 in the mRNA [11,33] (Fig 1C). In all four cell types, transfection of the A allele resulted in an RT-PCR product 480 bp larger than the canonical transcript encoding FL-hTERT, corresponding to the INS1b splice variant which we have previously shown by sequencing to result from retention of the first 480 bp of intron 4 [13] (Fig 1C). Other bands were sequenced and found to represent hybrid DNA species (Fig 1C).

We then genotyped a panel of cell lines to identify cell lines that were homozygous (AA) and heterozygous (GA) for the minor allele at rs10069690 (S1 Table). In order to determine the balance of FL-hTERT and INS1b splicing that occurs endogenously, a subset of the cell lines which were either homozygous or heterozygous for the minor allele at rs10069690 (S1 Table) were analyzed by RT-PCR. FL-hTERT was detected in all cell lines, while the levels of INS1 and INS1b transcript varied considerably (Fig 1D). MDA-MB-157 (AA) and B80-T8 (GA) cell lines displayed the highest levels of INS1b transcript, while HEK293T (AA) and BT549 (AA) had lower expression of INS1b. As the canonical splice donor site is retained in the presence of the SNP, FL-hTERT, INS1 and INS1b mRNA were all produced (Fig 1C and 1D). INS1b transcript was undetectable in CAOV3 (AA), MDA-MB-415 (AA), 27/87 (GA) and F80-T1 (GA).

The B80-T8 breast epithelial and F80-T1 fibroblast cell lines are genetically matched telomerase-positive cell lines derived from the same heterozygous individual [34,35], so it is of particular interest that the INS1b transcript is abundant in the breast cell line, whilst undetectable in the fibroblast cell line (Fig 1D). This indicates the potential for cell- or tissue-specific differences in INS1b generation, and suggests that complex regulatory mechanisms maintain the balance between INS1b and FL-hTERT.

Data from the 1000 Genomes Project demonstrated that rs10069690 is in linkage disequilibrium with another intron 4 SNP rs2242652 (r^2^ = 0.68) and an intron 3 SNP rs7725218 (r^2^ = 0.54). To investigate whether rs7725218 or rs2242652 influence hTERT splicing together or separately, we generated an hTERT minigene containing both intron 3 and intron 4, and incorporated various combinations of the major and minor alleles at rs7725218, rs2242652 and rs10069690 (S1A Fig). Minigenes were transfected into the MCF7 breast cancer cell line, and the 27/87 ovarian cancer cell line. RT-PCR was performed using primers spanning introns 3 and 4. No additional alternative splice variants were identified with any of these SNPs, when present in combination or individually (S1B and S1C Fig).

### Exogenous expression of INS1b in telomerase-positive cell lines inhibits growth rate

**Fig 2:**
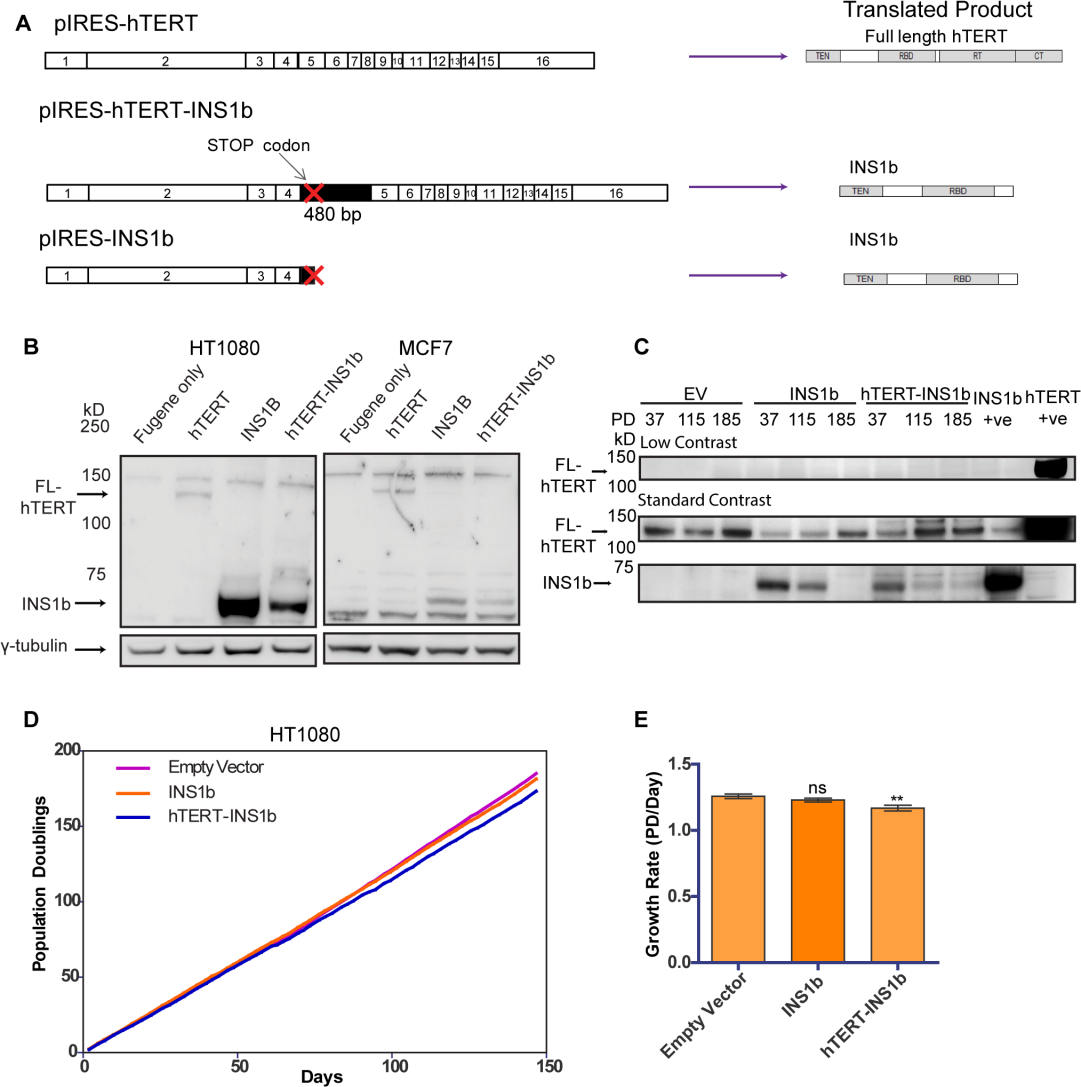
INS1b inhibits cell proliferation. (A) Schematics of expression constructs encoding the full-length (FL) hTERT protein only (pIRES-hTERT), and the hTERT-INS1b variant only (pIRES-hTERT-INS1b and pIRES-INS1b). (B) Western blot analysis of FL-hTERT, INS1b and γ-tubulin in HT1080 cells (left panels) and MCF7 cells (right panels) transfected with the constructs for 24 hours. (C) Western blot analysis of hTERT immunopurified from HT1080 cells stably transfected with pIRES empty vector (EV), pIRES-hTERT-INS1b and pIRES-INS1b. Samples from three different population doublings were blotted for FL-hTERT and INS1b. An hTERT immunopurified sample from pIRES-INS1b and pIRES-hTERT transfected 293T cells were used as positive controls. (D) Growth of stably transfected cell lines over 150 days. (E) Quantification of growth rates of each cell line calculated from time points every 2 to 3 days over 147 days (PD, population doubling; mean ± SEM; *P-value* calculated by two-tailed Student’s *t* test; ** p≤0.01).

The predicted hTERT INS1b protein lacks the reverse transcriptase domain, rendering it catalytically inactive. Therefore, we hypothesized that the relative amounts of FL-hTERT and INS1b in a cell directly determine telomerase activity levels. In order to investigate the function of INS1b, we disrupted the existing cellular balance of FL-hTERT and INS1b by exogenously expressing two different INS1b constructs that we expected would confer variable expression levels of the resulting proteins: a construct (pIRES-hTERT-INS1b) containing the complete cDNA of the alternatively spliced hTERT which consists of the FL-hTERT cDNA with the first 480 bp of intron 4 inserted, and another construct (pIRES-INS1b) which contains the hTERT-INS1b cDNA truncated at the stop codon in intron 4 (Fig 2A).

Variable levels of INS1b protein expression were consistently achieved with the pIRES-hTERT-INS1b and pIRES-INS1b constructs (Fig 2B), with greater expression being observed with the pIRES-INS1b construct. This was evident in multiple cell lines including HT1080 and MCF7, and following both transient and stable transfection (Fig 2B and 2C). Different expression levels were likely due to the relative sizes of the two constructs. The band detected by Western blot corresponding to the predicted size of INS1b (≈73 kD) was confirmed to be an hTERT variant by mass spectrometry analysis (S2 Fig).

To determine whether expression of INS1b had a direct effect on cell proliferation, growth curves were plotted for mass cultures of HT1080 cells stably expressing pIRES-hTERT-INS1b and pIRES-INS1b (Fig 2D and 2E). Protein expression was confirmed by Western blot analysis of immunopurified hTERT variants (Fig 2C). We observed robust expression of INS1b compared to endogenous FL-hTERT at early time points following stable transfection with pIRES-INS1b and pIRES-hTERT-INS1b, but not in the empty vector control. Interestingly, both the INS1b-expressing cultures spontaneously repressed the high INS1b to FL-hTERT protein ratios at later time points (Fig 2C), indicating a substantial selection pressure for FL-hTERT over INS1b. A small, but significant, reduction in growth rate was observed in the cell culture expressing pIRES-hTERT-INS1b compared to the empty vector control (Fig 2E). Overall, however, both INS1b expressing cell cultures maintained replicative capacity across 150 population doublings (pds) (Fig 2D).

These results were supported by the outcomes of transfecting the MCF7 cell line with the intron 4 minigenes containing either the major or minor alleles at rs10069690 (S3A Fig). A small, but significant, reduction in growth rate was observed with expression of the minigene containing the minor allele (A) (S3B and S3C Fig), consistent with the demonstrated production of INS1b transcript in this cell line (S3D Fig). However, both transfected MCF7 lines were also able to maintain replicative capacity over 70 pds. Notably, we were unable to obtain cultures stably expressing INS1b from the pIRES-INS1b construct, which supports the conclusion that expression of high levels of INS1b provides a selective disadvantage to the cells.

### Exogenous expression of INS1b resulted in telomere shortening accompanied by an increased telomere-specific DNA damage response

**Fig 3:**
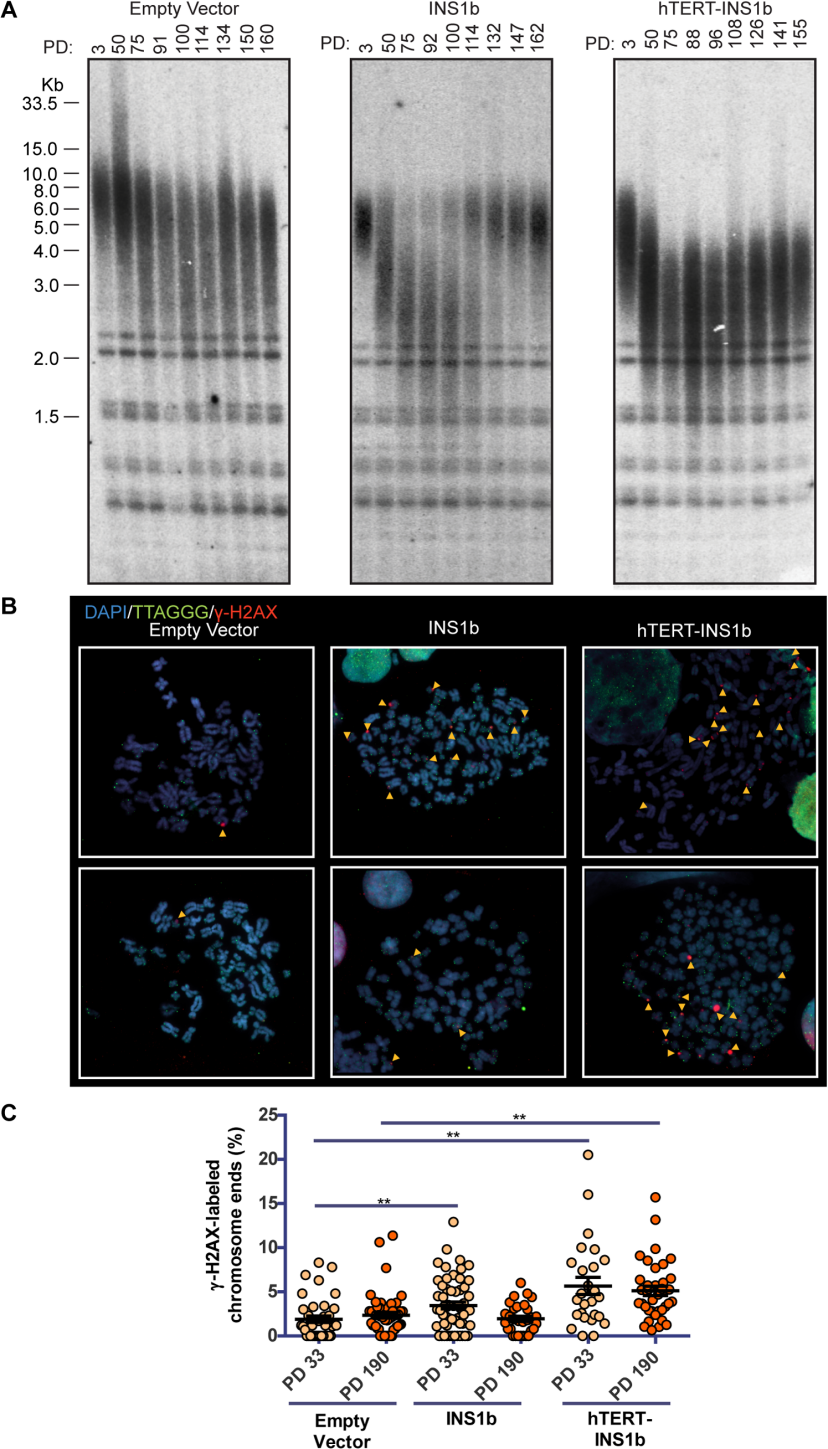
INS1b overexpression results in shorter telomeres and increased telomeric DNA damage response. (A) Terminal restriction fragment (TRF) length analysis of stably-transfected HT1080 cells at increasing population doublings (PD). (B) Metaphase telomere dysfunction-induced foci (Meta-TIF) analysis of stably-transfected HT1080 cells at PD33 (top) and PD190 (bottom). Metaphase cells were stained using γ-H2AX immunofluorescence (red), telomere FISH (green) and DAPI (blue). Meta-TIFs are indicated by yellow arrow heads. (C) Quantification of meta-TIFs calculated by the percentage of chromosome ends stained with γ-H2AX at one or both chromatids in each metaphase spread (mean ± SEM; *P-value* calculated by two-tailed Student’s *t* test; ** p≤0.01, compared to Empty Vector control at PD33 or PD190).

To determine whether altering the ratio of INS1b to FL-hTERT affects telomere length, we carried out terminal restriction fragment (TRF) length analysis on HT1080 cells overexpressing pIRES-INS1b or pIRES-hTERT-INS1b. Telomere length was measured approximately every 20 pds for a total of 160 pds (Fig 3A). Telomere length decreased slightly over this time course in the cultures expressing empty vector control, presumably due to gradual telomere length drift as has been observed previously in the HT1080 cell line [36]. In contrast, striking telomere shortening was observed during the first approximately 80 pds in both INS1b-expressing HT1080 cell cultures.

Telomere length was either restored (pIRES-INS1b) or stabilized (pIRES-hTERT-INS1b) at the later time points (Fig 3A). The mechanism underlying telomere length rescue appears to differ between the two cultures. In the pIRES-INS1b culture, the TRF pattern indicates that the small residual population of cells with long telomeres which was present at early pds eventually overgrew the cell population with short telomeres and dominated the culture at later pds. In the pIRES-hTERT-INS1b culture, telomeres underwent rapid shortening initially, but there was no further shortening after 70 pds (on the contrary, there was a small, gradual increase in length). However, even at the latest time points, telomere length did not fully recover and telomeres were substantially shorter than those of the empty vector or early pd cultures. Telomere length rescue coincided with the suppression of INS1b and restoration of FL-hTERT protein levels (Fig 2C).

To determine whether telomere shortening induced by INS1b overexpression resulted in an elevated telomere-specific DNA damage response (DDR), we quantified metaphase telomere dysfunction-induced foci (TIFs) on cytocentrifuged chromosomes from HT1080 empty vector, pIRES-INS1b and pIRES-hTERT-INS1b cell cultures at early (pd 33) and late (pd 190) timepoints (Fig 3B and 3C). An increase in TIFs was found to accompany telomere shortening in cells overexpressing INS1b. The number of TIFs was inversely correlated with telomere length.

To confirm the effects of the minor allele (A) on telomere length, TRF analysis was carried out on MCF7 cell cultures expressing the minigene constructs pIRES-hTERT Intron 4 Major (G) and pIRES-hTERT Intron 4 Minor (A) (S3E Fig). We utilized the minigene constructs to analyze telomere length changes in a more physiologically relevant system in which splice site utilization determines the balance between full-length and INS1b levels. The intron 4 Major (G) minigene generated FL-hTERT but no INS1b transcript (S3D Fig) and caused substantial telomere lengthening (S3E Fig; compare "Parental" lane to PD1). However, no telomere lengthening was observed with expression of the intron 4 minigene containing the minor (A) allele despite this construct producing both full-length and INS1b transcripts (S3D and S3E Fig). Telomere shortening was not observed, indicating that the balance of FL-hTERT and INS1b produced by this minigene is sufficient for telomere maintenance, but insufficient to support telomere lengthening.

### INS1b decreases telomerase activity by competitively binding to hTR to produce an inactive enzyme complex

**Fig 4:**
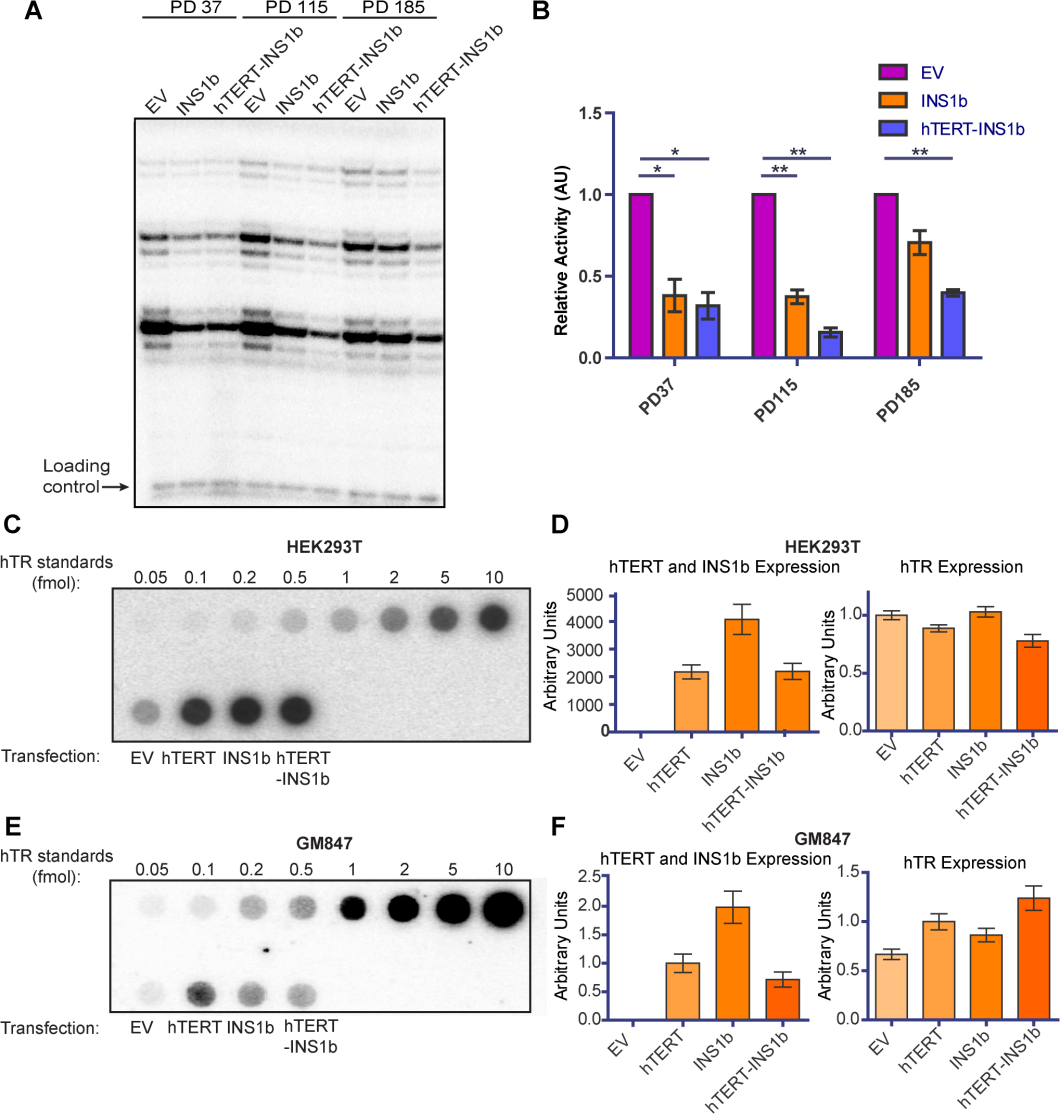
INS1b binds hTR and results in reduced telomerase activity. (A) Direct telomerase activity assay on equal amounts of telomerase immunopurified from HT1080 stable lines at three different population doublings (PD). (B) Quantification of the direct telomerase activity assay normalized to Empty Vector (EV) at each population doubling (mean ± SEM; n = 3 technical replicates; *P-value* calculated by two-tailed Student’s *t* test; *p≤0.05, ** p≤0.01). (C and E) Dot blots of recombinant hTERT and INS1b immunopurified with an anti-hTERT antibody from HEK293T cells (C) and GM847 cells (E) transfected with EV or an hTERT expression construct, and known amounts of *in vitro*-synthesized hTR (hTR standards), hybridized with a radiolabeled probe for hTR. (D and F) qRT-PCR analysis of hTERT, INS1b and hTR expression in transfected cell samples.

To investigate the mechanism by which expression of INS1b causes a decrease in telomere length, we examined the effect INS1b expression has on overall telomerase activity levels. We conducted direct telomerase activity assays on HT1080 cell cultures expressing pIRES-neo empty vector, pIRES-INS1b and pIRES-hTERT-INS1b at three different pds (Fig 4A). Telomerase activity was significantly depleted in both INS1b-expressing cell cultures at early and middle time points, compared to their empty vector counterparts (Fig 4B). At the PD185 time point, telomerase activity in the pIRES-INS1b culture had recovered to a level that was not significantly different from the empty vector, which correlated with full telomere length recovery. Telomerase activity remained low in the pIRES-hTERT-INS1b culture where telomere lengths initially decreased and then stabilized or recovered slightly. Consequently, both telomerase activity and telomere length recovery correlated positively with FL-hTERT protein levels and negatively with INS1b protein levels, directly demonstrating an inhibitory role for INS1b in telomerase-mediated telomere maintenance.

INS1b is predicted to retain the hTERT N-terminal RNA binding domain, but lacks the C-terminal reverse transcriptase domain. To directly determine whether INS1b is able to bind to hTR, FL-hTERT protein and INS1b were immunopurified following their transient co-expression with hTR in the HEK293T telomerase-positive cell line and the GM847 telomerase-negative cell line. GM847 cells do not express endogenous hTERT. Dot blot analysis to detect immunoprecipitated hTR demonstrated that INS1b was able to bind to hTR in both HEK293T and GM847 cells (Fig 4C and 4E). The observation that INS1b binds hTR in GM847 cells in the absence of FL-TERT indicates that binding occurs directly, rather than indirectly by dimerization with FL-TERT protein. Quantification of hTR, FL-hTERT and INS1b expression levels by qRT-PCR demonstrate relative expression of these telomerase components (Fig 4D and 4F).

It has previously been reported that hTERT possesses telomerase-independent functions, including mitochondrial polymerase activity, involvement in apoptosis, as well as a potential role in the Wnt signaling pathway [37–40]. A recent report, however, failed to substantiate a consistent role for hTERT in promoting the expression of Wnt target genes in human cell lines [41]. To investigate whether INS1b displayed non canonical functions in Wnt signaling, we transiently overexpressed the minigene constructs pIRES-hTERT Intron 4 Major (G) and pIRES-hTERT Intron 4 Minor (A), as well as pIRES-INS1b in MCF7 cells and compared expression of Wnt pathway genes to the pIRES-neo empty vector control using Wnt signaling PCR arrays. No activation of Wnt signaling genes was detected with FL-hTERT, low level INS1b expression from the minigene construct, or with the high levels of INS1b overexpression achieved from the cDNA construct (S4 Fig). These data do not support a role for hTERT or INS1b in the transcriptional modulation of Wnt target genes in these cells. Overall, our data demonstrate that INS1b functions as a dominant negative inhibitor of telomerase activity, most likely by sequestering hTR in an inactive enzyme configuration.

### Promotion of endogenous INS1b production using antisense oligonucleotides decreases telomerase activity

**Fig 5:**
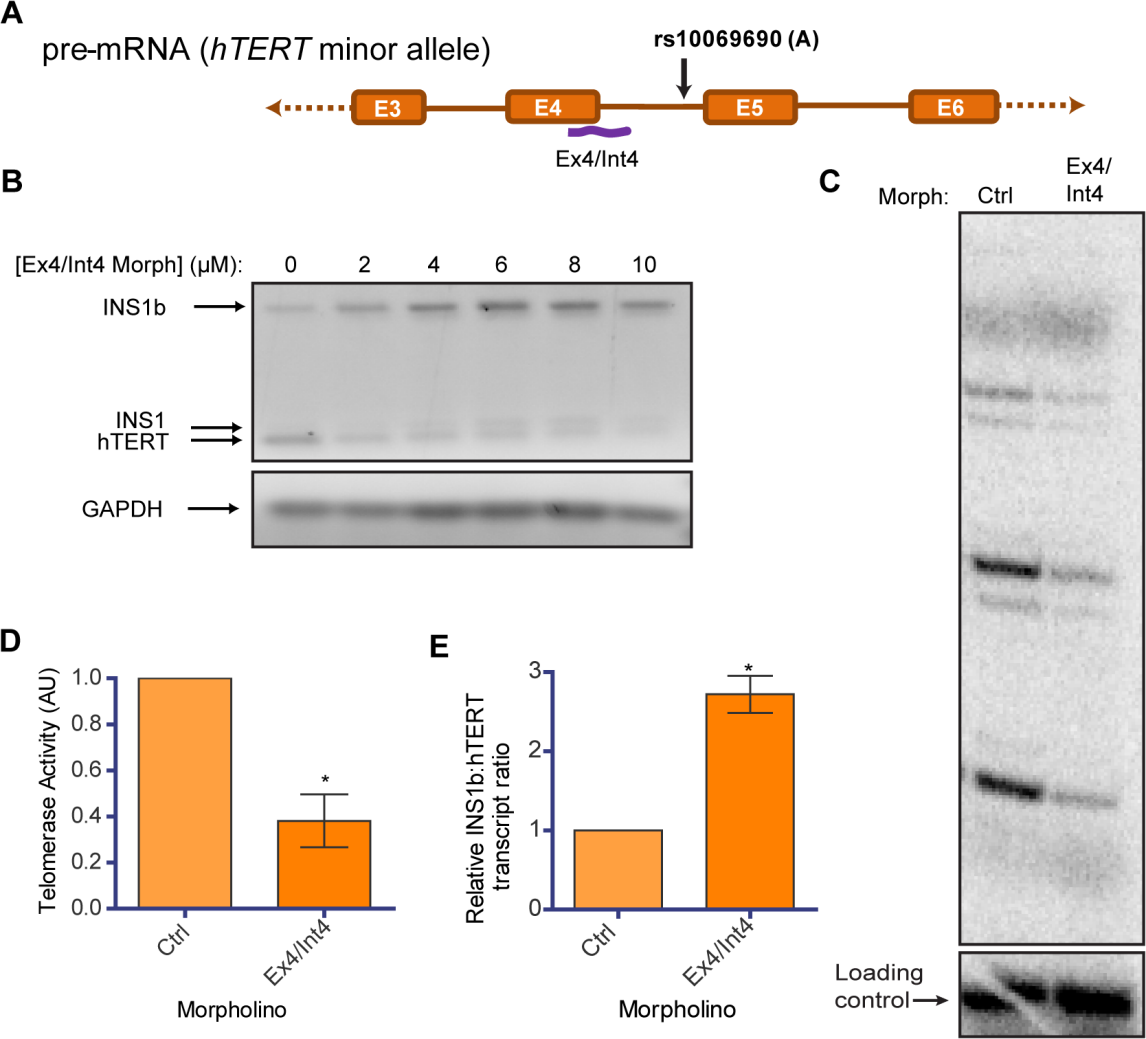
Endogenous modulation of hTERT intron 4 alternative splicing affects telomerase activity. (A) Schematic of position of binding of a morpholino (Ex4/Int4) (purple line) utilized to block splicing at the exon 4/intron 4 junction. (B) RT-PCR to determine concentration-dependent changes in INS1b and FL-hTERT transcript levels after delivery of the Ex4/Int4 morpholino into HEK293T cells. (C) Direct telomerase activity assay on immunopurified telomerase from equal numbers of HEK293T cells treated with 10 μM standard control (Ctrl) or Ex4/Int4 morpholino for 48 hours. (D) Quantification of the direct telomerase activity assay normalized to standard control morpholino (mean ± SEM; n = 3; *P-value* calculated by two-tailed Student’s *t* test; *p≤0.05). (E) Quantification of INS1b and FL-TERT expression after 10 μM morpholino treatment determined by RT-PCR on cells from each biological replicate of morpholino treatment. The INS1b:hTERT transcript levels ratio was calculated by densitometry and normalized to standard control morpholino (mean ± SEM; n = 3; *P-value* calculated by two-tailed Student’s *t* test; *p≤0.05).

Morpholino antisense oligonucleotides can be used to modify pre-mRNA splicing in the nucleus by targeting splice regulatory regions in a highly sequence specific manner [17,42]. To observe the effect of disrupting the balance of hTERT and INS1b endogenously, we designed a morpholino which bound and blocked the primary splice donor site at the exon 4/intron 4 boundary (Fig 5A). We delivered the morpholino (Ex4/Int4) into HEK293T cells, which are homozygous for the minor allele (A) at rs10069690, at a range of concentrations and performed RT-PCR 48 hours post-delivery. With increasing concentration, the morpholino inhibited FL-hTERT transcript production and enhanced INS1 and INS1b transcript levels (Fig 5B).

We conducted a direct telomerase activity assay using equivalent cell numbers to compare the Ex4/Int4 targeted morpholino to a standard non-targeting antisense oligonucleotide (ctrl), and observed a significant reduction in overall telomerase activity (Fig 5C and 5D). This directly demonstrates that endogenous telomerase activity can be inhibited by increasing the proportion of INS1b transcript to full-length transcript (Fig 5E), and indicates that manipulation of hTERT splicing may be a potential anti-telomerase therapeutic strategy, as proposed previously [43].

## Discussion

Candidate gene and genome-wide association studies have identified several SNPs in the *TERT-CLPTM1L* locus that are associated with cancer risk, and fine mapping has identified rs10069690 as a candidate causal SNP for breast and ovarian cancer risk. Currently, there are few direct functional studies that explain the biological mechanisms of such risk alleles, although a recent study has concluded that a substantial proportion of SNPs may modulate splicing in a tissue-specific way [44]. Here we demonstrate that the minor (A) allele of rs10069690 creates an additional, alternative splice donor site which results in the production of both FL-hTERT transcript and a variant transcript (INS1b) which has a premature stop codon and therefore encodes a severely truncated hTERT. This truncated INS1b protein lacks the reverse transcriptase domain, but maintains the hTR-binding domain, making it catalytically inactive and a dominant-negative inhibitor of telomerase activity. Other correlated SNPs (rs7725218 in intron 3 and rs2242652 in intron 4) were not found to alter hTERT splicing. Consistent with the association between rs10069690 and breast cancer risk, we found that the presence of the minor allele is associated with the presence of the INS1b transcript in telomerase-positive breast epithelial cells, but not in telomerase-positive fibroblasts from the same individual, suggesting that the balance between the two transcripts is subject to cell type-specific regulatory mechanisms.

We used overexpression studies to demonstrate that INS1b protein is produced, retains its hTR-binding capability, and inhibits telomerase activity determined by a quantitative, direct telomerase activity assay. Long-term overexpression of INS1b from two different cDNA expression constructs resulted in telomere shortening, which was accompanied by a telomere-specific DDR. This is most likely due to a dominant negative effect of the INS1b protein, because telomerase exists as a dimer [20] and its catalytic activity requires both hTERT active sites to be functional. It has previously been shown that disruption of the catalytic pocket in one of the two subunits exerts a dominant negative effect [20]. Therefore, even low levels of INS1b are likely to cause substantial effects on telomerase activity by dimerizing with active FL-hTERT molecules and sequestering hTR in catalytically inactive telomerase enzyme complexes. Recent studies have reported associations between minor alleles of rs2736100 [45,46] and rs7726159 [47] and longer telomeres in leukocytes, however population studies did not identify an association between the minor allele at rs10069690 and short mean telomere length [13]. It should be noted that the telomere length measurements were done in surrogate cells (i.e., peripheral blood cells rather than breast or ovarian epithelial cells), and that any effects of physiological levels of INS1b on telomere length in normal cells are likely to be more subtle than the effect of overexpressing this variant transcript in cancer cells.

Multiple hTERT splice variants have been identified at variable, and often abundant, levels in telomerase-positive cancer cell lines [48]. As with INS1b, the vast majority involve truncations of the C-terminus and generate catalytically inactive products [12,49,50]. The abundant hTERT β-deletion splice variant, which results from a 183 nucleotide deletion between exons 6 and 9, was found to be translated into a truncated protein that competed for binding to hTR and inhibited endogenous telomerase activity [19]. Nevertheless, cancer cell lines are able to proliferate rapidly in the presence of abundant inactive splice variants. The mean telomere length of most tumors is shorter than that of the surrounding normal tissue, suggesting that a relatively short, optimal telomere length is selected for and maintained in tumor cells, possibly to decrease the replicative burden presented by longer telomeres. It is possible that expression of alternative splice variants is selected for in telomerase-positive tumors as one means of preventing excessive telomere lengthening from occurring. Conversely, we observed that telomerase-positive cell lines prevented excessive telomere shortening as a result of long-term INS1b overexpression by down-regulating expression of INS1b from the cDNA constructs, via mechanisms that we did not examine, consistent with the notion that there is selection for an optimal telomere length in cancer cells.

Minigene constructs that included intron 4 with each allele at rs10069690 were used to study effects of the additional splice site in a more physiological manner than the cDNA overexpression constructs allowed [51]. Over-expression of FL-hTERT caused telomere lengthening in HT1080 cells [36,52], and this occurred as expected with the minigene containing the major allele, which expresses FL-hTERT but not INS1b. In contrast, we identified a telomere-lengthening defect conferred by the presence of the risk-associated minor (A) allele which co-expresses both FL-hTERT and INS1b. Unlike the INS1b cDNA constructs which encode only INS1b, the minigene did not lead to a telomere shortening phenotype, further supporting the conclusion that the relative levels of INS1b and FL-hTERT affect telomerase activity and telomere length.

By characterizing endogenous INS1b in cell lines homozygous and heterozygous for the minor allele at rs10069690, we identified highly variable levels of INS1b relative to FL-hTERT across different cell lines. Most strikingly, we observed high levels of endogenous INS1b expression in the breast epithelial cell line B80-T8, whereas there was no detectable INS1b expression in the genetically matched fibroblast cell line F80-T1. This demonstrates that there are clear differences in regulation of hTERT splicing between different cell types, including cells isolated from different tissues of the same individual. Indeed, it has previously been shown that differential alternative splicing of hTERT transcripts occurs non-randomly in different tissue types during human development [15]. Differences in splicing may arise from alterations in the chromatin environment encompassing rs10069690, which may render differences in binding of splicing regulatory factors and favor the use of a particular splice site. This is supported by our previous study, in which we used site-specific formaldehyde-assisted isolation of regulatory elements (FAIRE) analysis of an approximately 1 kb region spanning rs10069690, and identified open chromatin signatures in breast stromal and myoepithelial/stem cell samples, whereas closed chromatin signatures were observed in progenitor and differentiated luminal epithelial cell fractions [13].

Finally, to demonstrate the effect of endogenous INS1b we introduced morpholino antisense oligonucleotides designed to sterically block FL-hTERT splicing which resulted in the preferential use of the alternative rs10069690 splice donor site, shifting the balance of transcripts in a dose-dependent manner in favor of INS1b. This resulted in decreased telomerase activity, demonstrating the inhibitory effects of increasing proportions of INS1b transcript in the absence of any exogenous expression constructs. Modulation of hTERT splicing is therefore a potential therapeutic strategy for limiting (or, by inhibiting alternative splicing, increasing) telomerase activity [10,17].

**Fig 6:**
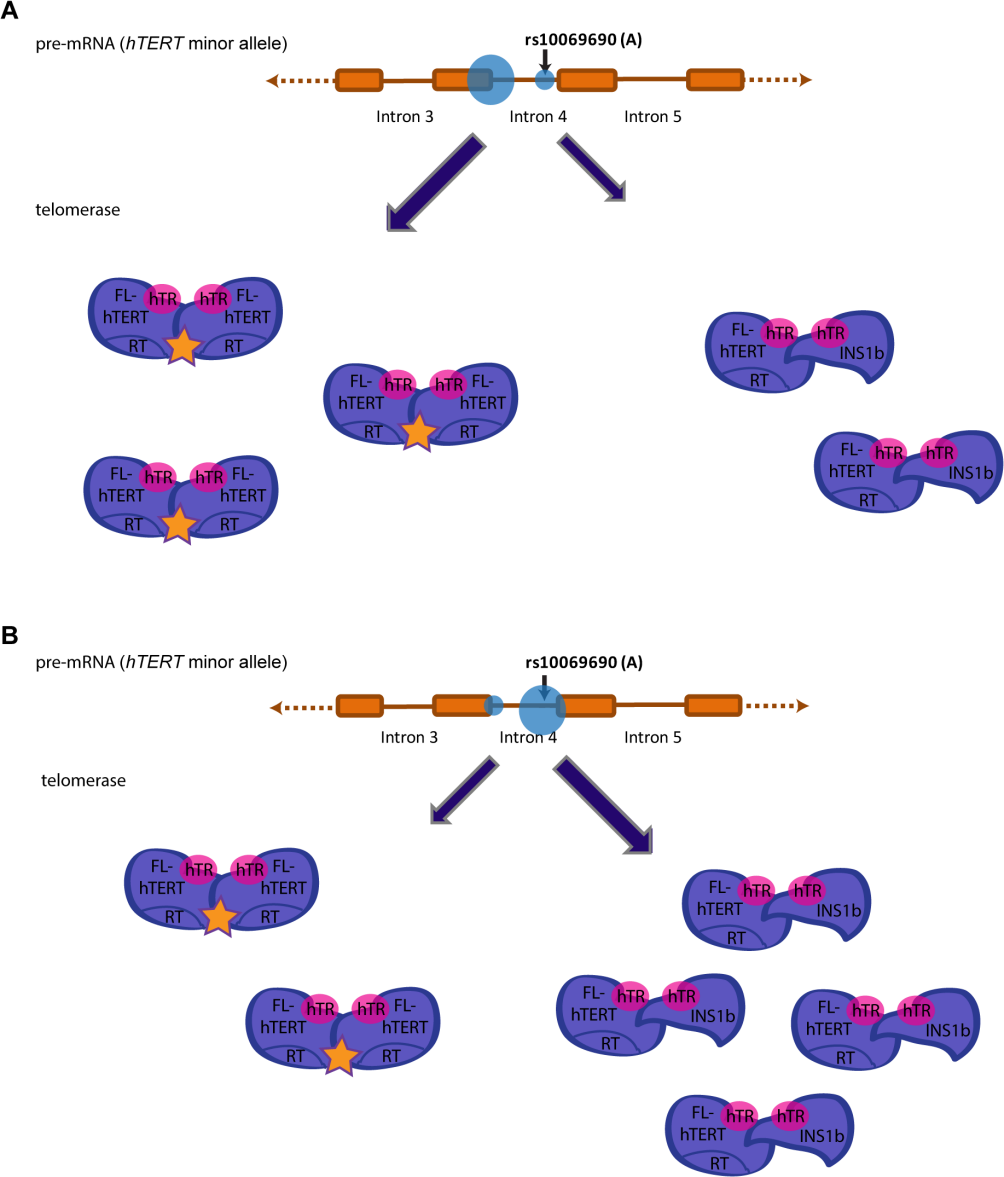
Preferential splicing at the donor site generated by the minor allele (A) at rs10069690 reduces overall telomerase activity. Splicing events at intron 4 of hTERT in individuals that possess the minor allele (A) at rs10069690 could alter telomerase activity levels. (A) Preferential splicing at the exon 4/intron 4 junction would yield a higher proportion of FL-hTERT to INS1b, resulting in a greater proportion of telomerase dimers assembled with two FL-hTERT molecules and hence more active telomerase complexes. (B) Preferential splicing at the additional splice donor site created by the A allele would yield a lower proportion of FL-hTERT to INS1b. This would result in more dimers consisting of at least one INS1b molecule which does not have a reverse transcriptase domain, and therefore a higher proportion of catalytically inactive telomerase complexes. Hence, preferential splicing at this site would reduce overall telomerase activity.

In summary, we provide the first evidence that a well-documented cancer risk-associated polymorphism in the *TERT-CLPTM1L* locus encodes an alternative splice variant of hTERT, which results in a catalytically inactive telomerase enzyme complex (Fig 6). Because hTERT and hTR levels are both limiting, this results in decreased telomerase activity and limits telomere lengthening, consistent with a dominant negative effect. The balance between INS1b and FL-hTERT levels is critical, and appears to be regulated in a cell-specific manner. We suggest a model whereby expression of INS1b is of functional relevance during development in tissue-specific stem cell populations in which telomerase activity is limiting. Subtle defects in telomerase-mediated telomere extension may result in shorter telomere lengths, and will cause cells to reach proliferative exhaustion or replicative senescence, earlier than in individuals without the risk allele. The increased risk of genetic instability ensuing from shorter telomeres is predicted to increase the risk of tumorigenesis. Cancer risk may be further increased in the context of BRCA1 mutations, which suppress DNA repair mechanisms [53]. This is consistent with the minor allele at rs10069690 conferring additional breast cancer risk in BRCA1 mutation carriers [13]. Therefore, the minor allele at rs10069690 creates an avenue for regulation of telomerase activity where it can increase the risk of cancer-predisposing short telomeres in normal adult tissues and contribute to optimal telomere maintenance in tumor cells.

## Materials and Methods

### Experimental design

**Fig 7:**
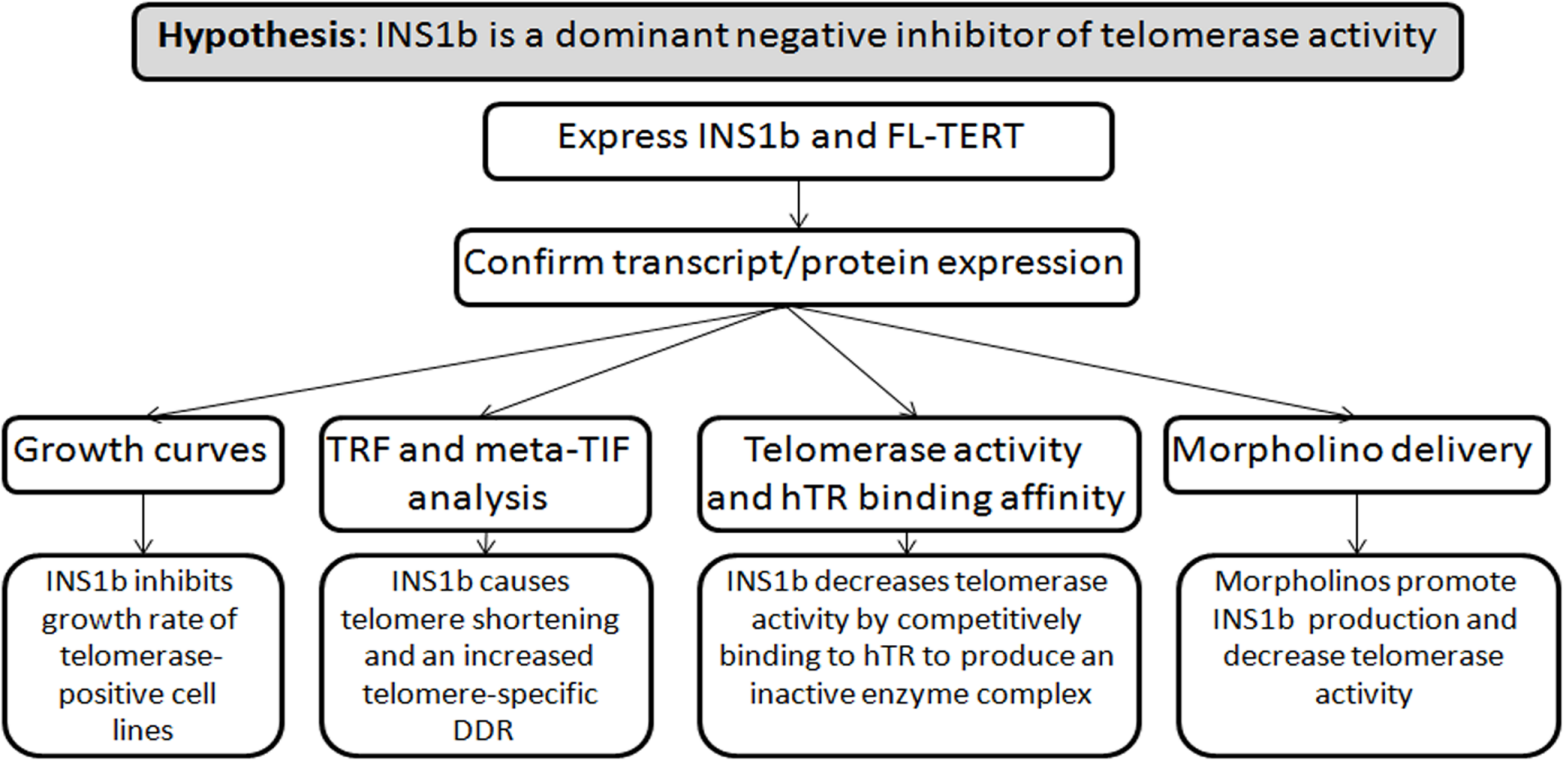
Experimental design diagram. Summary of hypothesis and experimental approach.

Summary of experimental design (Fig 7).

### Plasmid and minigene construction

pIRES-hTERT [18], pIRES-hTERT Intron 4 Major (G), pIRES-hTERT Intron 4 Minor (A), pIRES-hTERT Intron 4-rs2242652 (A) and pIRES-hTERT Intron 4-(A)(A) (minor alleles at both sites) were developed from pIRES-neo (Clontech) as described previously [13]. A construct containing the first 480 bp of intron 4 of hTERT in a pUC57 vector (GenScript) was subcloned into pIRES-hTERT to generate pIRES-hTERT-INS1b. To generate a plasmid (designated pIRES-INS1b) containing the translated sequence of INS1b, this region was PCR amplified from pIRES-hTERT Intron 4 Major (G) and cloned into pIRES-neo.

Site directed mutagenesis (Agilent Technologies) was used to introduce the minor allele at rs7725218 into pUC57 intron 3 (GenScript). Each version of intron 3 and fragments of pIRES-hTERT Intron 4 Major (G) and pIRES-hTERT Intron 4(A)(A) were then PCR amplified and ligated using the In-Fusion HD Cloning Kit (Clontech). The minigenes generated were designated pIRES-hTERT Intron 3(G)/Intron4(G)(G) (major alleles at all intron 3 and 4 sites), pIRES-hTERT Intron 3(G)/Intron 4(A)(A) (minor alleles at rs2242752 and rs10069690), pIRES-hTERT Intron 3 (A)/Intron 4(G)(G) (minor allele at rs7725218) and pIRES-hTERT Intron 3(A)/Intron 4(A)(A) (minor allele at all sites). All constructs were validated by sequence analysis prior to transfection.

### Cell culture

Cell lines were purchased from American Type Culture Collection (ATCC), with the exception of the B80-T8 and F80-T1 telomerase-positive cell lines [35] and the Fre-71s-1 cell strain [54] which were established by L. Huschtscha. Culture media and supplements were purchased from Invitrogen Life Technologies. MCF7, HEK293T, HT1080, MDA-MB-157, CAOV-3, Fre-71s-1 and F80-T1 cells were cultured in Dulbecco’s modified Eagle’s medium (DMEM) with 10% v/v Fetal Bovine Serum (FBS). MDA-MB-415 cells were cultured in DMEM with 15% FCS and 10 μg/mL insulin. A2780, BT549 and 27/87 cells were cultured in RPMI 1640 medium with 10% FBS. B80-T8 cells were cultured in a 1:1 ratio of RPMI 1640 and MCDB-170. All cells were maintained at 37°C and 5% CO_2_. Cell lines were authenticated by 16-locus short tandem repeat profiling and were confirmed to be free of *Mycoplasma* species by CellBank Australia (Children’s Medical Research Institute, Westmead, New South Wales, Australia).

### Transient overexpression of minigenes and plasmids

pIRES-hTERT Intron 4 Major (G) and pIRES-hTERT Intron 4 Minor (A) were transiently transfected into HT1080, MCF7, A2780 and Fre-71s-1 cells using siPORT NeoFX Transfection Agent (Life Technologies) and harvested after 24 hours for RT-PCR analysis.

For all other overexpression experiments, plasmid constructs were transfected into HEK293T, HT1080, MCF7 and GM847 cells using Fugene 6 Transfection Reagent (Promega) and harvested after 24–72 hours. Overexpression was confirmed using quantitative reverse transcription PCR (qRT-PCR) analysis.

### Stable overexpression of constructs and growth curve analysis

pIRES constructs were stably overexpressed in HT1080 and MCF7 cells, followed by 48 hours recovery and treatment with G418 at 700 μg/mL for HT1080 cells and 800 μg/mL for MCF7 cells. The surviving cells were grown to 95% confluency before initiating growth curve analysis. At this point the cell population was designated population doubling (pd) 1. To measure growth rates of each cell line, cells were counted and seeded at known numbers every 2 to 4 days while being maintained at exponential growth phase.

### Genotyping cell lines

Genomic DNA was genotyped using a custom Fluidigm 96 multiplex and SNP Type chemistry. Assays were designed by Fluidigm and performed according to the manufacturer’s protocol using a Fluidigm Biomark HD. To ensure quality control, genotyping cluster plots were inspected visually and samples which failed >20% assays were removed from analysis.

### Morpholino design and treatments

The morpholino antisense oligonucleotide “Ex4/Int4 Morph” (5'-TTAAACCAAAGCACAGCCACCCTCT-3') and Standard Control Oligonucleotide (5'CCTCTTACCTCAGTTACAATTTATA-3') were synthesized by Gene Tools. Concentrations of 2–10 μM of Ex4/Int4 Morph were delivered to HEK293T cells with 6 μL/mL Endo-Porter delivery reagent (Gene Tools) and cells were harvested after 48 hours.

### RNA extraction, cDNA synthesis and PCR analysis

Total RNA was extracted using the RNeasy Mini Kit (Qiagen) and DNase I digested (Life Technologies). cDNA was synthesized from 1–5 μg of RNA using the SuperScript III First-Strand Synthesis System (Life Technologies). hTERT and INS1b transcripts were amplified using primers spanning from the exon 3/4 junction to exon 6 (Intron 4 F1: 5'-GGAATCAGACAGCACTTGAAGAGGGT-3'; Intron 4 R3: 5'-TTTGATGATGCTGGCGATGACCTC-3') with Abgene Recombinant *Taq* DNA polymerase and Buffer (ThermoScientific), 2 mM MgCl_2_, 2 mM of each dNTP (Roche) and 10% dimethyl sulfoxide (DMSO) (Sigma) for 30 cycles. Intron 3 was amplified using primers spanning Exon 3 to Exon 4 (Intron 3 F1: 5'- AGATCCTGGCCAAGTTCCTGC-3'; Intron 3 R1: 5'-CGACGTAGTCCATGTTCACAATCG-3') and intron 4 amplified using primers spanning Exon 4 to Exon 6 (Intron 4 F4: 5'- ATTGTGAACATGGACTACGTCGTGGG-3'; Intron 4 R3: 5'- TTTGATGATGCTGGCGATGACCTC-3'). For quantitative transcript measurements, GAPDH transcripts were amplified (GAPDH F: 5'- ACCCACTCCTCCACCTTTG-3'; GAPDH R: 5'-CTCTTGTGCTCTTGCTGGG-3') under the same conditions, without DMSO, for 21 cycles. The products were resolved on a 1.3% agarose gel with 500 ng/mL ethidium bromide alongside NEB 100 bp DNA ladder and visualized by the Alpha Innotech FluorChem 5500 system. Quantification was performed by densitometry using ImageQuant software. To quantify overexpression after transient transfection with hTERT, INS1b and hTR constructs, qRT-PCR was performed using SYBR Green (Roche) with LightCycler 96 (Roche) according to the manufacturer’s instructions. One primer set was used to measure both FL-hTERT and INS1b overexpression (MS TERT F: 5'-AAGTTCCTGCACTGGCTGATGAGT-3', MS TERT R: 5'-CACCCTCTTCAAGTGCTGTCTGAT-3') and a another to quantify hTR (hTR F: 5'-CTAACCCTAACTGAGAAGGGCGTA-3', hTR R: 5'- GGCGAACGGGCCAGCAGCTGACATT-3').

### Western blots

Whole cell lysates or telomerase immunoprecipitations were separated by SDS-PAGE and Western Blotting performed using the NuPAGE system (Invitrogen) as per manufacturer’s instructions. Primary antibodies used include HTCS2 sheep anti-TERT [55] at 0.5 μg/mL for INS1b and overexpressed FL-hTERT detection, Rockland rabbit anti-telomerase catalytic subunit diluted 1:500 in PBS-T (8 mM Na_2_HPO_4_, 150 mM NaCl, 2 mM KH_2_PO_4_, 3 mM KCl, 0.1% Tween 20) for endogenous hTERT detection [56] and Sigma rabbit anti-tubulin diluted 1:500. The secondary antibodies used were rabbit anti-sheep immunoglobulin horseradish peroxidase (Dako) diluted 1:2000 and goat anti-rabbit immunoglobulin horseradish peroxidase (Dako) diluted 1:5000.

### Immunopurification of FL-hTERT and INS1b

FL-hTERT and INS1b were immunopurified using an hTERT antibody (HTCS2), as described previously [57].

### hTR binding of FL-hTERT and INS1b

Dot blotting of immunopurified FL-hTERT and INS1b samples with probe against hTR was performed as described previously [58].

### Direct telomerase activity assay

The direct telomerase activity assay was performed on immunopurified telomerase normalized by hTR amount or cell number as described previously [57].

### Terminal restriction fragment (TRF) analysis

Terminal restriction fragment (TRF) analysis to determine telomere length was performed as described previously [36].

### Metaphase telomere dysfunction-induced foci (Meta-TIF) analysis

Meta-TIF analysis was performed as described previously [59] with the exception that after cytocentrifugation the cells were permeabilized in pre-extract buffer (20 mM HEPES, 20 mM NaCl, 5 M MgCl_2,_, 300 mM Sucrose, 0.5% v/v NP40) with gentle shaking, washed once in PBS-T and washed twice in PBS before fixing in PBS with 4% v/v formaldehyde.

### Wnt signaling PCR array analysis

MCF7 cells were harvested 48 hours after transfection with pIRES-neo, pIRES-hTERT Intron 4 Major (G), pIRES-hTERT Intron 4 Minor (A) and pIRES-INS1b, RNA was extracted using the RNeasy Mini Kit (Qiagen) and cDNA was synthesized using the RT^2^ First Strand Kit (Qiagen). The cDNA samples were then loaded on to qPCR human Wnt signaling PCR arrays (PAHS-043YF-2) and relative transcript levels of each gene measured by qPCR on LightCycler 96 (Roche) according to the manufacturer’s instructions. Results were analyzed on the RT^2^ Profiler PCR Array Data Analysis pathway software (SABiosciences).

### Mass spectrometry analysis

hTERT and INS1b immunopurified samples were prepared and separated on a 4–12% NuPAGE Novex Bis-Tris Mini Gel (Life Technologies). The gel was stained with Coomassie Staining Solution (0.0025% w/v coomassie brilliant blue G, 45% methanol and 10% glacial acetic acid) overnight at room temperature and washed with destaining solution (45% methanol and 5% glacial acetic acid) for 4–6 hours. The gel was rehydrated with two 15 min washes in water and bands corresponding to the sizes of hTERT (125 kD) and INS1b (73 kD) proteins excised. Gel pieces were rinsed with three 10 min washes in water. They were then destained at 37°C in wash solution (50% v/v acetonitrile, 12.5 mM NH_4_HCO_3_ pH 7.8) and dehydrated using GeneVac EZ-2 plus. To digest the proteins at lysine residues, 15 ng/μl trypsin (sequencing grade; Promega) in 25 mM NH_4_HCO_3_ pH 7.8 was added directly to the gel pieces and, after they became translucent, 25 mM NH_4_HCO_3_ pH 7.8 was added to cover the gel pieces. Samples were incubated at 37°C overnight with shaking. They were then briefly spun down, 0.5% v/v trifluoroacetic acid (TFA) was added to cover gel pieces and samples were incubated in a water bath sonicator for 15 min. Samples were loaded onto activated Eppendorf GELoader tips with filter plugs (3M Empore C18 filter membrane), washed in 0.5% v/v TFA and eluted in 70% v/v acetronitrile and 0.5% v/v TFA solution. Samples were spotted onto an Opti-TOF 384 Well Insert with equal volumes of matrix (70% v/v acetonitrile, 0.5% TFA). The insert was analysed by MS/MS on a MALDI-TOF/TOF AB SCiex 5800 system and protein identification performed by the Mascot program.

## Supporting Information

S1 Fig Other intronic single nucleotide polymorphisms associated with rs10069690 do not affect hTERT alternative splicing. (A) Schematics of hTERT minigene constructs with different permutations of alleles at rs7725218 in intron 3, rs2242752 in intron 4 and rs10069690. (B and C) RT-PCR on MCF7 and 27/87 cells transfected with these constructs using primers spanning intron 3 (B) and intron 4 (C).
(TIF)

S2 Fig Confirmation that INS1b is an alternative splice variant of hTERT. FL-hTERT and INS1b were overexpressed and immunopurified from HEK293T cells. Immunopurified samples separated on a polyacrylamide gel and stained with Coomassie Solution. Bands corresponding to FL-hTERT (127 kD) and INS1b (73 kD) were excised and prepared for mass spectrometry analysis using the MALDI-TOF/TOF system. The 127 kD and 73 kD bands were confirmed by mass spectrometry to contain hTERT peptide sequences with high Mascot scores.
(TIF)

S3 Fig The minor allele at rs10069690 confers defective telomere lengthening in MCF7 cells. (A) Schematics of hTERT intron 4 minigene constructs with each allele at rs10069690 and the potential proteins produced. (B) Growth curve analysis of MCF7 cells stably transfected with the minigene constructs. (C) Quantification of growth rates of each cell line calculated from time points every 2 to 3 days over 100 days (mean ± SEM; *P-value* calculated by two-tailed Student’s *t* test; *p≤0.05). (D) RT-PCR analysis of FL-hTERT and INS1b levels in the stably-transfected lines over a range of population doublings. (E) Terminal restriction fragment (TRF) analysis of stable MCF7 cell cultures at increasing population doublings and the parental cell line.
(TIF)

S4 Fig Full-length hTERT and INS1b do not affect the Wnt signaling pathway. PCR array analysis of 84 human Wnt pathway genes in MCF7 cells transfected with hTERT intron 4 minigene and INS1b overexpression constructs for 48 hours. Results are plotted as a scatter plot where each point represents a gene; the x-axis is the empty vector control transcript levels and the y-axis is (A) the hTERT Intron 4 Major G allele, (B) the hTERT Intron 4 Minor A allele, and (C) the INS1b transfected sample transcript levels. Both axes are in logarithmic scale (n = 3).
(TIF)

S1 Table Genotypes of cell lines at rs10069690 and rs2242652. (PDF)
