# Supplementary figures and images for: A Common Cancer Risk-Associated Allele in the hTERT Locus Encodes a Dominant Negative Inhibitor of Telomerase
Source: PLoS Genet. 2015 Jun 8;11(6):e1005286. doi: 10.1371/journal.pgen.1005286 (PMC4459975; doi:10.1371/journal.pgen.1005286)

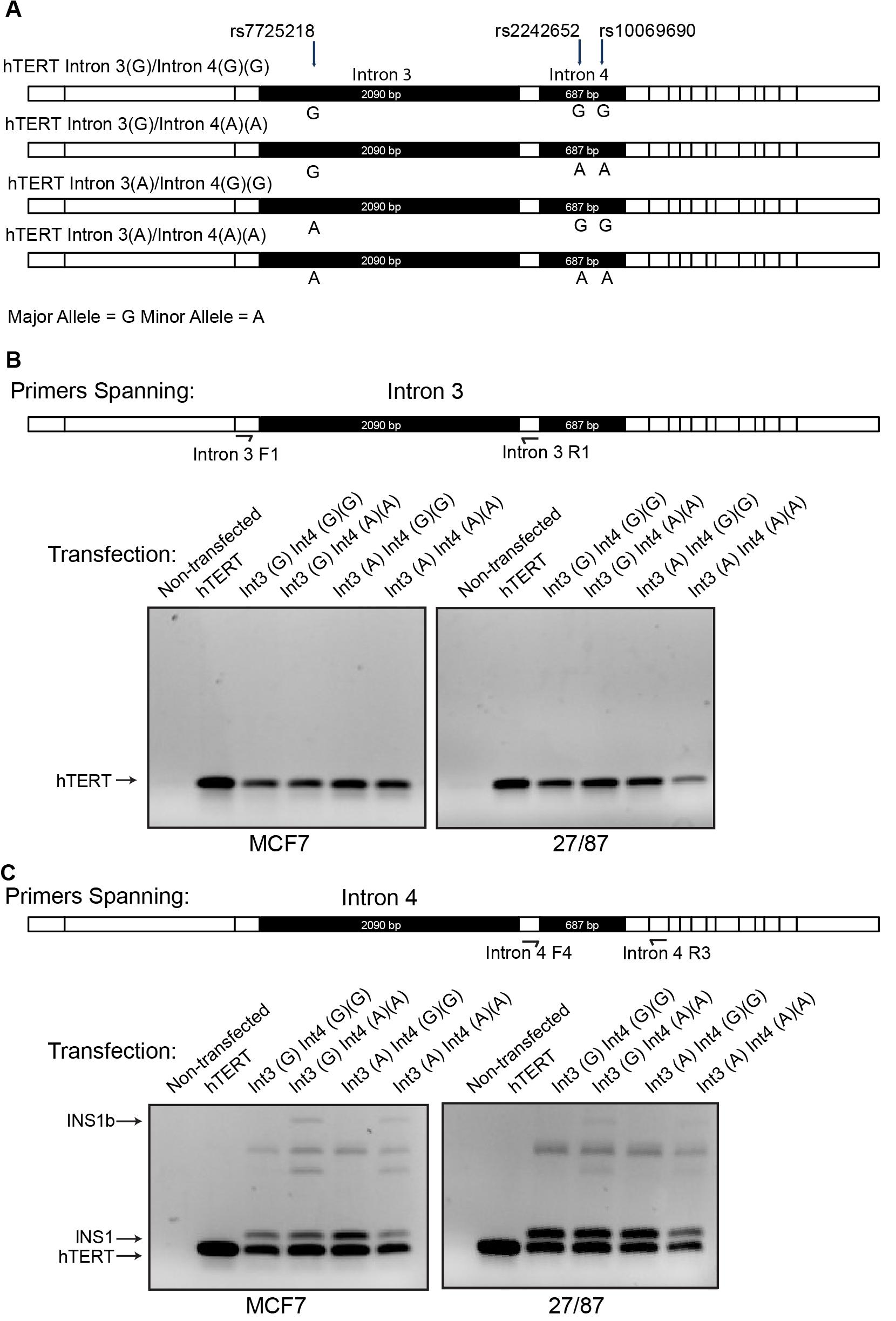

Supplement: S1 Fig — (A) Schematics of hTERT minigene constructs with different permutations of alleles at rs7725218 in intron 3, rs2242752 in intron 4 and rs10069690. (B and C) RT-PCR on MCF7 and 27/87 cells transfected with these constructs using primers spanning intron 3 (B) and intron 4 (C). (TIF) [file pgen.1005286.s001.tif]

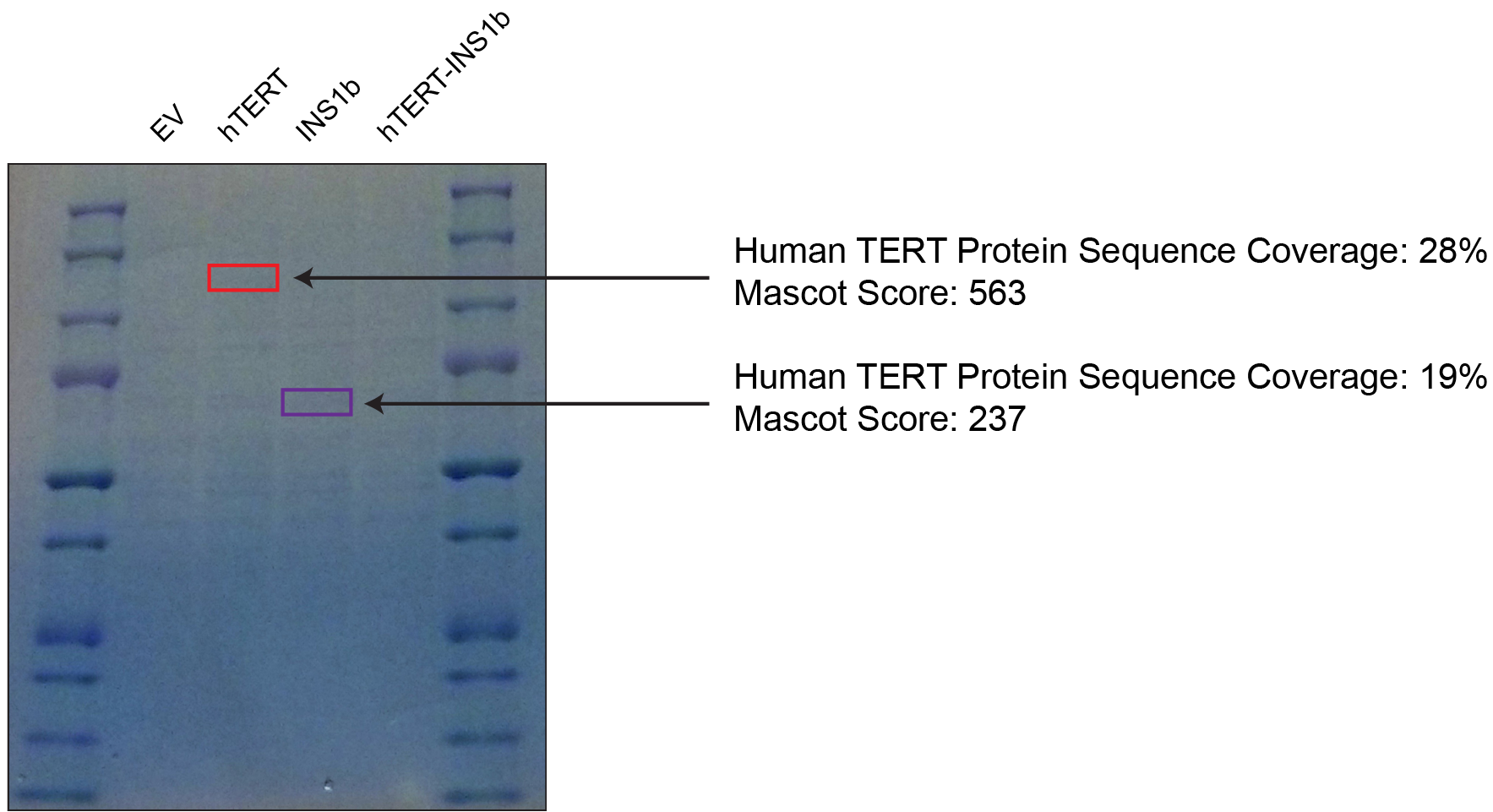

Supplement: S2 Fig — FL-hTERT and INS1b were overexpressed and immunopurified from HEK293T cells. Immunopurified samples separated on a polyacrylamide gel and stained with Coomassie Solution. Bands corresponding to FL-hTERT (127 kD) and INS1b (73 kD) were excised and prepared for mass spectrometry analysis using the MALDI-TOF/TOF system. The 127 kD and 73 kD bands were confirmed by mass spectrometry to contain hTERT peptide sequences with high Mascot scores. (TIF) [file pgen.1005286.s002.tif]

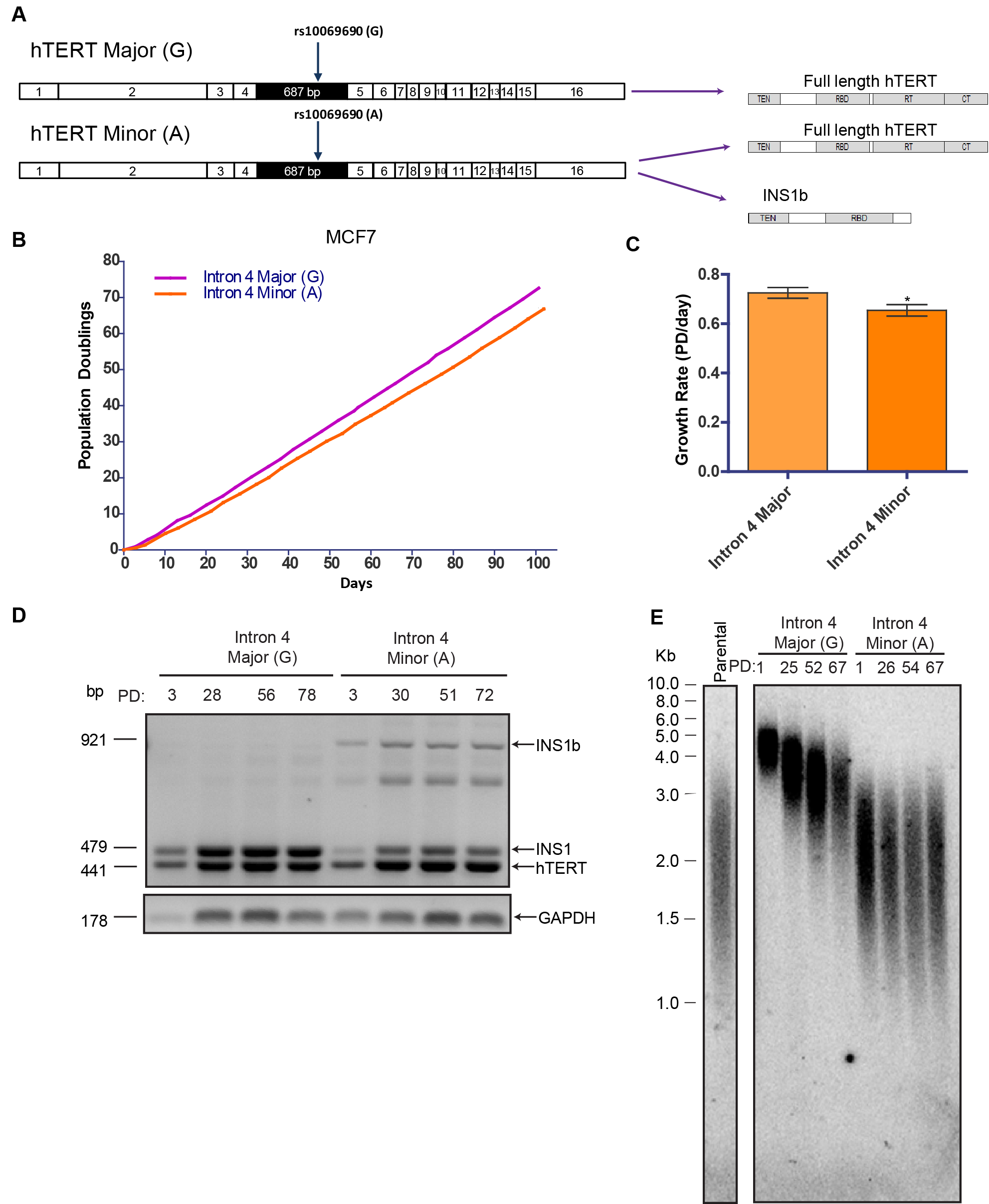

Supplement: S3 Fig — (A) Schematics of hTERT intron 4 minigene constructs with each allele at rs10069690 and the potential proteins produced. (B) Growth curve analysis of MCF7 cells stably transfected with the minigene constructs. (C) Quantification of growth rates of each cell line calculated from time points every 2 to 3 days over 100 days (mean ± SEM; P-value calculated by two-tailed Student’s t test; *p≤0.05). (D) RT-PCR analysis of FL-hTERT and INS1b levels in the stably-transfected lines over a range of population doublings. (E) Terminal restriction fragment (TRF) analysis of stable MCF7 cell cultures at increasing population doublings and the parental cell line. (TIF) [file pgen.1005286.s003.tif]

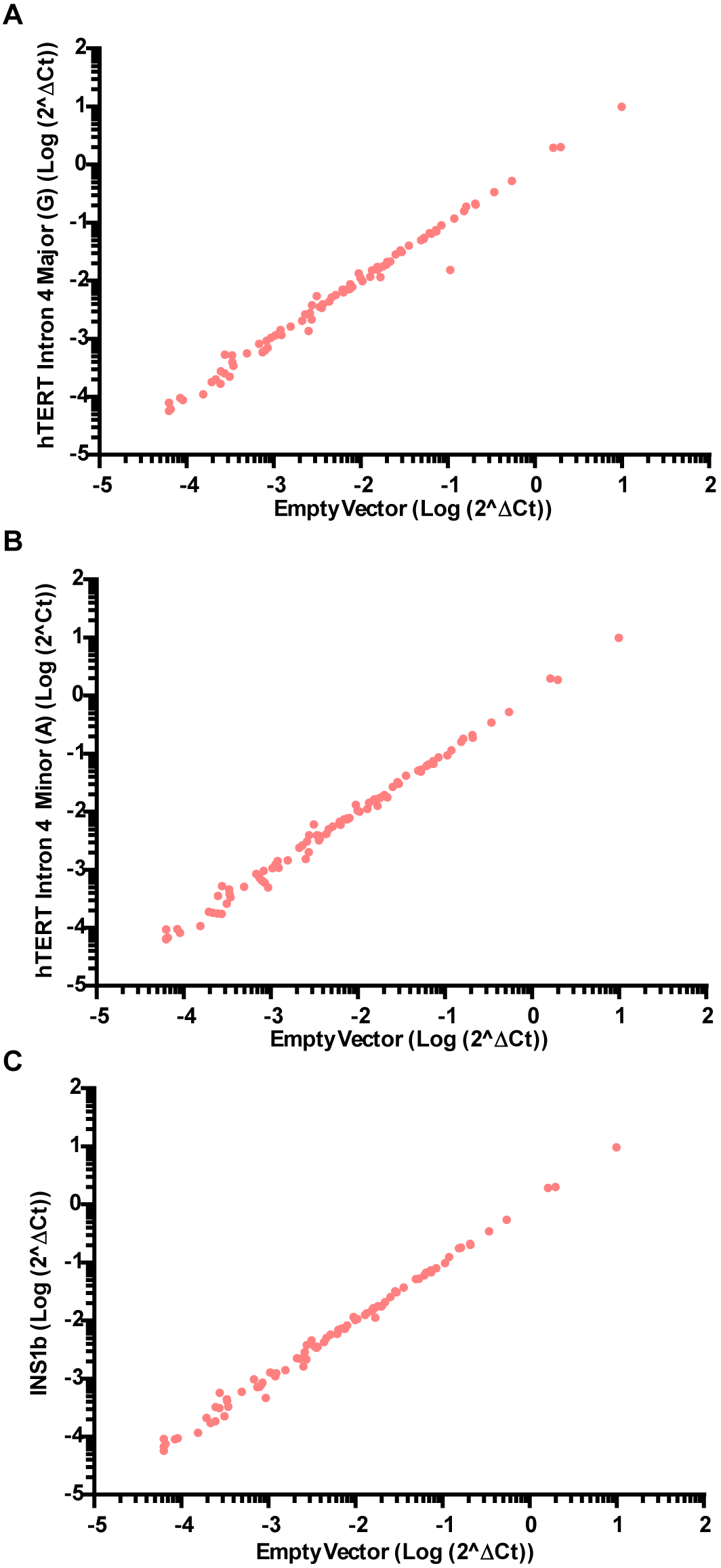

Supplement: S4 Fig — PCR array analysis of 84 human Wnt pathway genes in MCF7 cells transfected with hTERT intron 4 minigene and INS1b overexpression constructs for 48 hours. Results are plotted as a scatter plot where each point represents a gene; the x-axis is the empty vector control transcript levels and the y-axis is (A) the hTERT Intron 4 Major G allele, (B) the hTERT Intron 4 Minor A allele, and (C) the INS1b transfected sample transcript levels. Both axes are in logarithmic scale (n = 3). (TIF) [file pgen.1005286.s004.tif]
