# Supplementary material for: A Common Cancer Risk-Associated Allele in the hTERT Locus Encodes a Dominant Negative Inhibitor of Telomerase
Source: PLoS Genet. 2015 Jun 8;11(6):e1005286. doi: 10.1371/journal.pgen.1005286 (PMC4459975; doi:10.1371/journal.pgen.1005286)
Supplement: S1 Table — (PDF) [file pgen.1005286.s005.pdf]

Supplementary Table 1

Cell Line	rs10069690 Haplotype	rs2242652 Haplotype	Origin Tissue
184A1	G:A	G:A	Breast
184B5	G:A	G:A	Breast
27/87	G:A	G:G	Ovary
8701BC	A:A	-	Breast
A2780	G:G	G:G	Ovary
B80-hTERT	G:A	G:A	Breast
BT474	G:A	G:A	Breast
BT549	A:A	G:A	Breast
CAOV3	A:A	A:A	Ovary
CaOV4	G:G	G:G	Ovary
COLO 316	G:A	G:A	Ovary
ES2	G:A	G:G	Ovary
HaCaT	G:G	G:G	Keratinocyte
HCC1143	G:G	G:G	Breast
HCC1937	A:A	G:A	Breast
HCC1954	A:A	G:G	Breast
HCC70	A:A	G:G	Breast
HEK 293T	A:A	A:A	Kidney
HeLa	G:G	G:G	Cervix
HEY	A:A	G:A	Ovary
HOSE 17.1	G:A	G:G	Ovary
HOSE 6.3	G:A	G:A	Ovary
HS578T	G:A	G:G	Breast
IGROV1	G:G	G:G	Ovary
Ishikawa	G:A	G:A	Uterus
MCF 10A	G:G	G:G	Breast
MCF7	G:G	G:G	Breast
MDAH-2774	G:A	G:G	Ovary
MDA-MB-134	G:G	G:G	Breast
MDA-MB-157	A:A	G:G	Breast
MDA-MB-175-VII	A:A	G:A	Breast
MDA-MB-231	G:G	G:G	Breast
MDA-MB-330	G:G	G:G	Breast
MDA-MB-361	G:G	G:G	Breast
MDA-MB-415	A:A	A:A	Breast
MDA-MB-436	G:A	G:G	Breast
MDA-MB-453	G:A	G:A	Breast
MDA-MB-468	G:A	G:G	Breast
OAW42	G:G	G:G	Ovary
OVCAR3	G:G	G:G	Ovary
PEO1	G:G	G:G	Ovary
PEO14	G:G	G:G	Ovary
PEO4	G:G	G:G	Ovary
PMC-42 ET	G:G	G:G	Breast
SBOT 3.1	G:A	G:G	Ovary
SK-BR-3	G:A	G:G	Breast
SKOV3	G:A	G:A	Ovary
SUM-159-PT	G:G	G:G	Breast

SVCT	G:A	G:A	Breast
T-47D	G:G	G:G	Breast
UACC-812	G:A	G:G	Breast
ZR-75-1	G:G	G:G	Breast
